# PHYSICIANS’ PERCEPTIONS OF AGE-FRIENDLY CARE IMPLEMENTATION FOR OLDER ADULTS LIVING WITH CANCER

**DOI:** 10.1093/geroni/igae098.2937

**Published:** 2024-12-31

**Authors:** Kylie Sloan

**Affiliations:** University of California San Francisco, San Francisco, California, United States

## Abstract

This qualitative pilot study investigates physicians’ perceptions about the implementation of age-friendly care (e.g., 4Ms framework) within an Age-Friendly Health System (AFHS), with a specific focus on care for older adults living with cancer. The pilot uses a critical grounded theory approach to conduct and analyze virtual, semi-structured, one-on-one interviews with 6 physicians, recruited using purposive snowball sampling between October 2023-March 2024. Participants currently provide care to older adults with diagnosed cancer and work in an AFHS. Half the participants are geriatricians and the remaining have no geriatric training. All are between ages 30 and 60, 4 identify as White, 1 as Asian, 1 as biracial (Asian and White), 5 as female, 1 as male, and all worked at their organization for between 6 and 15 years. Three preliminary findings emerged: 1) Participants offer differing definitions for the “age” in “age-friendly” and look beyond a number for who to consider an “older” adult; 2) Participants (without geriatric training) express limited knowledge about AFHS and suggest making the health system’s physical and digital spaces more accessible for older patients with cancer; 3) Participants indicate implementation of some “M’s” (e.g., What Matters, Medication) more than other “M’s” (e.g., Mentation, Mobility) within cancer care. Money, time, and staffing are key barriers to age-friendly care implementation in cancer care according to participants. Findings inform potential gaps in age-friendly care implementation (e.g., depression screening/referral process) to address within cancer care and future research exploring perceptions of older patients with cancer about age-friendly care.

